# Circular RNAs: a rising star in respiratory diseases

**DOI:** 10.1186/s12931-018-0962-1

**Published:** 2019-01-05

**Authors:** Jian Wang, Mengchan Zhu, Jue Pan, Cuicui Chen, Shijin Xia, Yuanlin Song

**Affiliations:** 1 Department of Pulmonary Medicine, Zhongshan Hospital, Fudan University, 180 Fenglin Road, Shanghai, 200032 China; 2 Department of Infectious Diseases, Zhongshan Hospital, Fudan University, 180 Fenglin Road, Shanghai, 200032 China; 3 Department of Geriatrics, Shanghai Institute of Geriatrics, Huadong Hospital, Fudan University, 221 West Yan An Road, Shanghai, 200040 China

**Keywords:** Circular RNAs, Non-coding RNA, miRNA sponge, Alternative splicing, Respiratory diseases

## Abstract

Circular RNAs (CircRNAs), as a new class of non-coding RNA molecules that, unlike linear RNAs, have covalently closed loop structures from the ligation of exons, introns, or both. CircRNAs are widely expressed in various organisms in a specie-, tissue-, disease- and developmental stage-specific manner, and have been demonstrated to play a vital role in the pathogenesis and progression of human diseases. An increasing number of recent studies has revealed that circRNAs are intensively associated with different respiratory diseases, including lung cancer, acute respiratory distress syndrome, pulmonary hypertension, pulmonary tuberculosis, and silicosis. However, to the best of our knowledge, there has been no systematic review of studies on the role of circRNAs in respiratory diseases. In this review, we elaborate on the biogenesis, functions, and identification of circRNAs and focus particularly on the potential implications of circRNAs in respiratory diseases.

## Background

In the early 1970s, Sanger et al. [1] detected the presence of circRNAs in the plant viroid for the first time by electro-microscopy. Soon after, similar circular RNAs were found in yeast mitochondria and human hepatitis D virus [2, 3]. Nigro et al. [4] detected the generation of circular transcripts in their study of the tumor suppressor gene DCC, which was the first confirmation of the existence of circRNAs in human cells. However, due to the limitations of research methods that existed at the time, circRNAs were disregarded as transcription artifacts or splicing noise, without any important role in biological processes. With the advent of next-generation sequencing technology, coupled with bioinformatics, large numbers of circRNAs have been detected across various species from archaea to humans. Many of these circRNAs are abundant, endogenous, stable and conserved [5–7], with specificity based on the species, tissues, diseases, and developmental stages of the organism [8–10]. Now, there is considerable interest in circRNAs in the field of RNA research.

**Fig. 1:**
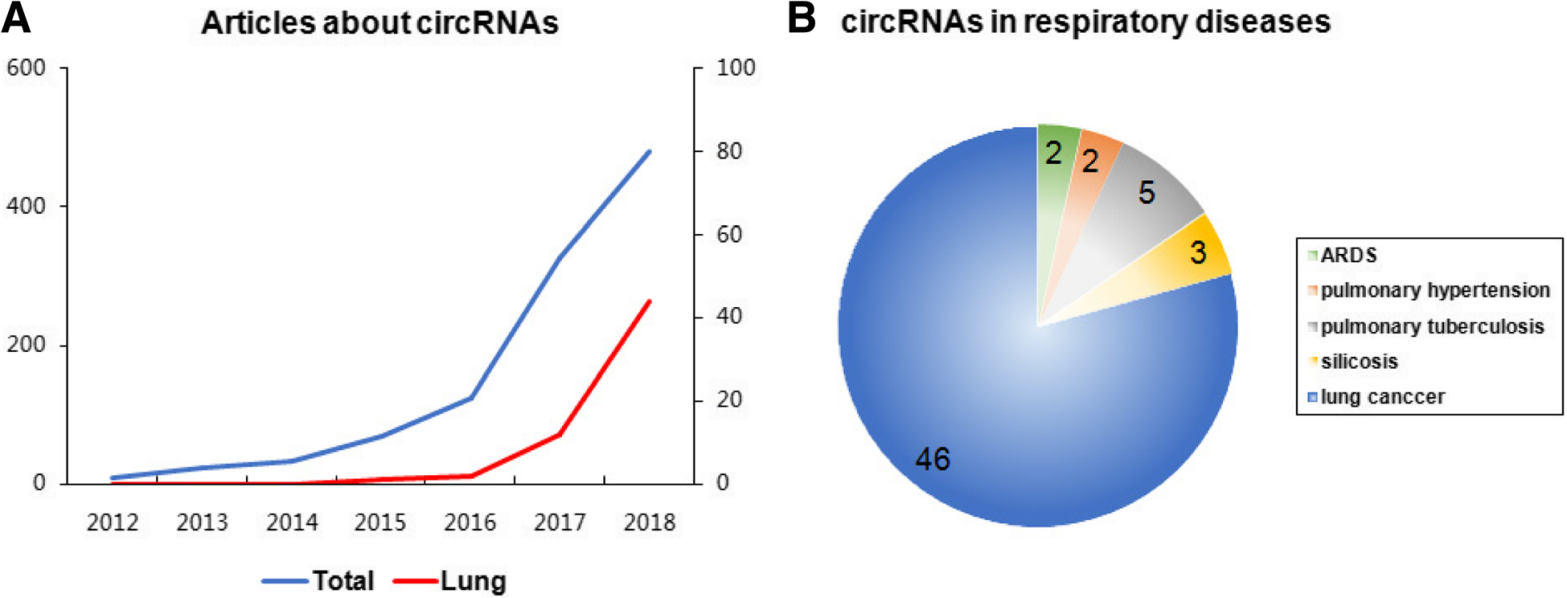
The number of articles about circRNAs in Pubmed. **a** Respective amounts of published articles about circRNAs in total (blue line) or on the lung (red line) in PubMed. Left Y axis is for blue line and Right Y axis is for red line. **b** Respective amounts of published articles about circRNAs on different respiratory diseases in PubMed

Up until September 17, 2018, we identified 1370 records about “circular RNA”, “circRNA” or “RNA, circular” in PubMed (https://www.ncbi.nlm.nih.gov/pubmed/). The number of total publications and publications with a focus on the lung increased year by year. Among them, a dramatic increase has present since the year of 2017, which suggests the promising prospect of circRNAs in the diagnosis and treatment of human diseases, including respiratory diseases (Fig. 1). Respiratory diseases are some of the most common medical conditions in the world and are considered as one of the leading causes of global mortality [11]. Recent studies have identified a lot of differential circRNAs in different respiratory diseases using the circRNAs microarray or the next-generation sequencing. Furthermore, the biological mechanisms of several circRNAs in pathologic processes of some respiratory diseases have also been revealed [12–14]. To the best of our knowledge, our team elucidated the expression profile of dysregulated circRNAs in the lung of mice with hypoxia-induced pulmonary hypertension (PH) for the first time [15]. However, so far, no comprehensive review or summary of researches on the role of circRNAs in different respiratory diseases were conducted, even though some reviews have provided a brief introduction [16, 17]. Thus, we have generalized the biogenesis, functions and identification of circRNAs, with a particular focus on respiratory diseases.

### How circRNAs are formed

**Fig. 2:**
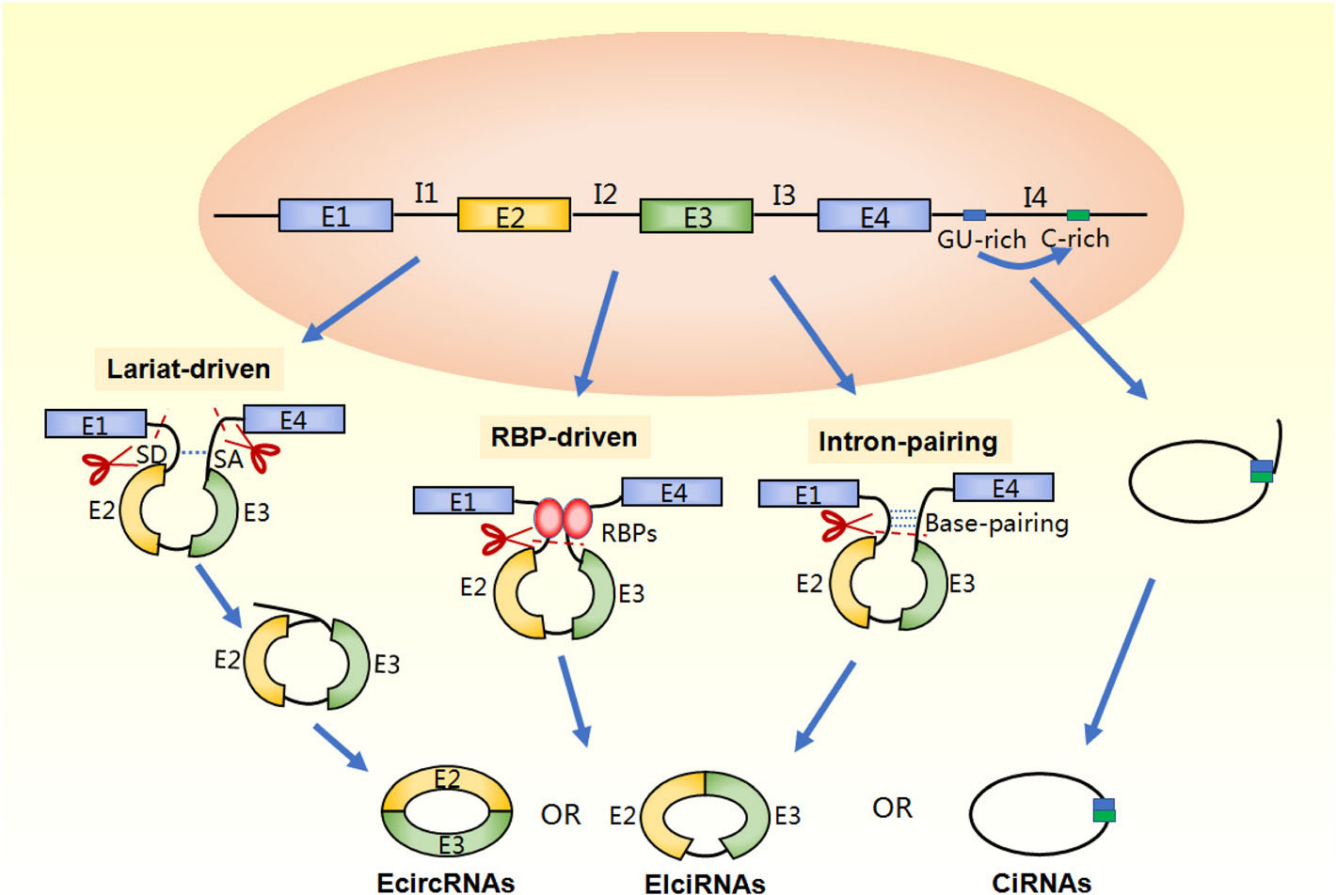
Formation of exonic circRNAs (EcircRNAs), exon-intron circRNAs (EIciRNAs), and intronic circRNAs (CiRNAs). Two models of EcircRNAs and EIciRNAs formation exist, including lariat-driven circularization and intron-pairing-driven circularization, which can be catalyzed by complementary sequences and RNA-binding proteins (RBPs). CiRNA formation depends mainly on consensus motifs near both ends

The mechanisms of circRNA formation, including those of exonic circRNAs (EcircRNAs), intronic circRNAs (CiRNAs), and exon-intron circRNAs (EIciRNAs), have been gradually revealed through progressive research (Fig. 2). Like canonical (linear) splicing, back-splicing requires both a canonical splicing signal and the canonical spliceosome machinery, where a downstream splice donor site (5′ splice site) is spliced to an upstream acceptor splice site (3′ splice site) in reverse order [18, 19].

So far, two well-known models of EcircRNA formation have been proposed, namely the ‘direct back-splicing’ model and the ‘exon skipping’ model [7, 20–22]. The main difference between the two models is the order in which of the two processes comes first, canonical splicing or back-splicing. In the direct back-splicing model, as the name suggests, back-splicing happens first, and the two introns are paired with complementary motifs, and the 5′ splice donor and the 3′ splice acceptor are juxtaposed to generate EcircRNAs directly. In the exon skipping model, canonical splicing happens first, and exon skipping occurs in the precursor mRNAs (pre-mRNAs) to form a lariat that contains exons. Splicing of the exons before and after the lariat by canonical splicing results in the formation of mature linear RNA, while the excised lariat is then back-spliced to generate EcircRNAs. During the biogenesis of the EcircRNAs, introns may not be spliced out completely but retained between the encircled exons in the newly generated circRNAs. This phenomenon was reported by Li et al. [23] in mammalian cells, and the RNAs were refered to as EIciRNAs, a special subtype of circRNAs. Unlike EcircRNAs and EIciRNAs, the formation of CiRNAs in karyocytes depends mainly on the 7-nucleotide GU-rich motif located at the 5′ splicing site and the 11-nucleotide C-rich motif at the 3′ branch site. During back-splicing, the two elements bind into a lariat-like intermediate, which contains the excised exons and introns, and are cut out by the spliceosome. The stable lariats thereby generated then undergo 3′ tail degradation, which results in the formation of CiRNAs. The unique method by which CiRNAs are formed results in distinct differences from EcircRNAs; CiRNAs are 2′-5′ phospholipid-linked molecules, and EcircRNAs are 3′-5′ phospholipid-linked molecules [24].

Furthermore, back-splicing is considered to be extensively regulated by canonical cis-acting splicing regulatory elements and trans-acting splicing factors. Zhang et al. [25] found that splicing selection and exon circularization are regulated by complementary sequences within or across individual flanking introns. Complementary sequences within individual flanking introns can be sufficient to promote linear mRNA generation, while that across flanking introns can benefit exon circularization. Trans-factors, such as RNA binding proteins (RBPs), reportedly serve a regulatory role in circRNAs biogenesis. For example, Quaking, which play a role in pre-mRNA splicing, binds at a specific sequence on the intron, so that both ends of the loop fragment on the RNA precursor are close to each other and finally form a circRNA by reverse splicing [26]. The MBL protein can bind to the conserved sequence on both the exonic and flanking intronic sequences of MBL pre-mRNA, regulate its own precursor RNA to form circRNAs, and further link the conserved sequence of MBL to a minigene that can sufficiently induce circRNAs biogenesis [27]. Khan et al. [28] also found that RBM20 protein knockout significantly inhibited the formation of myosin complexes. In contrast, a negative correlation between ADARs (Adenosine deaminases acting on RNA) and circRNAs biogenesis has also been reported. The silencing of ADAR1 increases the expression of intracellular circRNAs, and the mechanism by which this occurs possibly is associated with the ability of ADAR1 to melt the dsRNA hairpin structure when inverted complementary sequences are present in the flanking introns of circularized exons [29].

### How circRNAs work

**Fig. 3:**
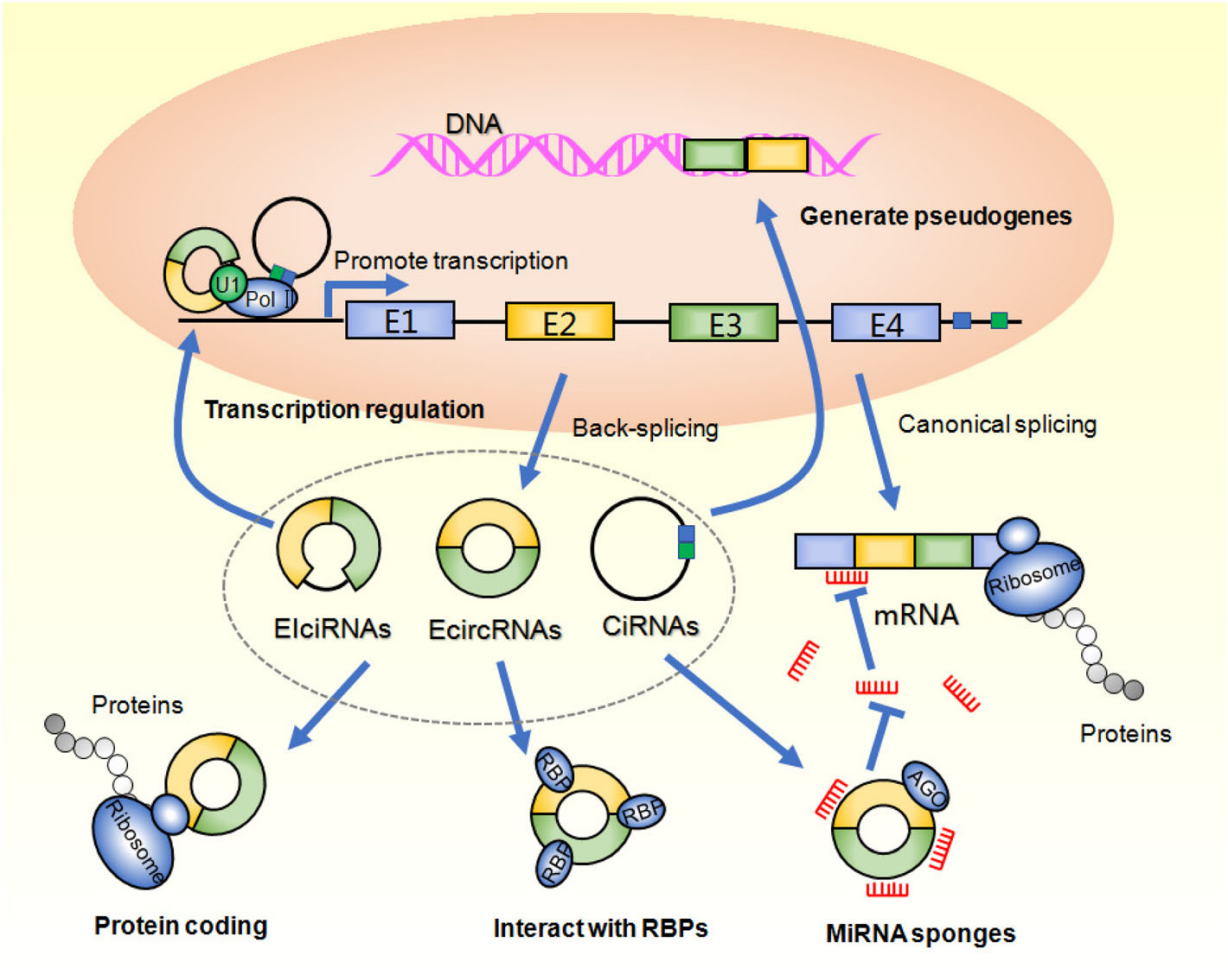
Biological functions of circRNAs. CircRNAs act as miRNA sponge to decrease the expression level of miRNA via miRNA response elements and further to regulate the expression of downstream mRNA. CircRNAs could regulate the transcription of parental genes and adjust its variable splicing. CircRNAs could combine with RBPs to regulate the post-transcriptional process. CircRNAs could directly encode protein. CircRNAs-derived pseudogenes could retrotranscribed and reinserted into the host genome

With an increasing number of in-depth research studies on circRNAs, more functions of circRNAs have been revealed over just the last several years (Fig. 3).

#### Super sponge for microRNAs

MicroRNAs (miRNAs) with a size of ~ 20 nt, post-transcriptionally regulate the translation of target mRNAs via binding miRNA response elements (MREs). The studies have provided evidences that some of circRNAs harbor MREs, suggesting a potential role as competitive endogenous RNAs (ceRNAs) that compete for miRNA-binding sites, thereby affecting miRNA activities [30]. In 2013, two different study teams simultaneously demonstrated for the first time that circRNAs function as powerful miRNAs sponges [31, 32]. Both teams found overlapping co-expression of ciRS-7/CDR1as and miR-7 in nerve tissue, suggesting their close interactions in vivo. Other studies have shown that cyclic RNA ciRS-7/CDR1as harbors more than 70 conventional miR-7 binding sites, and thus function as a super-sponge of miR-7. In addition, Hansen et al. [32] found that mouse testicular-specific circRNA (Sry) also function as a sponge that binds to miR-138. The ability of circRNAs’ miRNA-binding capacity is stronger than that of any other ceRNAs; they are therefore known as “super sponges”.

#### Transcriptional and post-transcriptional regulation, and alternative splicing

EIciRNAs and CiRNAs localize predominantly in the nucleus and are currently shown to regulate the transcription of parental genes. It has been found that EIciRNAs, such as circEIF3J and circPAIP2, can bind to U1 small nuclear ribonucleoproteins (snRNPs) to form a complex, and then interact with RNA polymerase II in the promoter region of the parental gene to enhance transcription of that gene [33]. Similarly, CiRNAs interact with RNA polymerase II in the nucleus and regulate host transcription in a cis-acting manner [24].

During the process of precursor RNA splicing, back-splicing can compete to adjust the variable splicing. Ashwal-Fluss et al. [18] discovered that the second exon of the splicing factor MBL could undergo cyclization to form circMbI. The flanking intron of circMbI has a conserved MBL binding site that binds closely to MBL. Changing the expression level of MBL can affect the expression of circMbI, whereas the expression of circMbI changes the competitive regulation of canonical pre-mRNA splicing and thus affects the expression of MBL. Another study confirmed that the formation of myosin circulatory cells in RBM20 knockout mice was significantly reduced, whereas the exon of the myosin circadian antibody was increased in the linear mRNA, suggesting that the process of circRNAs synthesis regulates the process of variable splicing. Many other circRNAs that contain the translation-initiation site possess similar function and act as an mRNA trap to regulate expression of their host genes.

In addition, circRNAs bind to RBPs in a sponge-adsorbed form and are capable of storing, sorting and transferring RBPs, thereby inhibiting their function and regulating the post-transcriptional process [31].

#### Protein coding

The function of circRNAs encoding proteins was first demonstrated in the hepatitis D virus [34]. However, in eukaryotes, Chen et al. [35] demonstrated that circRNAs with internal ribosome entry sites (IRES) could be efficiently translated in vitro. In addition, Wang et al. [36] constructed a miRNA that contains the cytomegalovirus promoter, IRES, and the exon of the GFP, which can be channeled into the cell to form a circRNA. This circRNA can also translate the GFP protein, as evidenced by green fluorescence in the cells. These studies indirectly demonstrate that circRNAs play a role in coding proteins, even though they may be lacking in direct evidence. The three latest studies have directly confirmed that circRNAs in eukaryotic cells can encode proteins. Legnini et al. [37] found that circ-ZNF609 had an open reading frame (ORF), both ends of which contain the start and end codes, respectively, that can transduce the polynucleotide to the protein. Pamudurti et al. [38] defined a group of circRNAs associated with translational ribosomes in Drosophila using ribosome imprinting. Mass spectrometry showed that a circRNA from a blind muscle site could encode a protein. In addition, Yang et al. [39] found that circRNAs were extensively translated by N6-methyladenosine. However, to date, no direct evidence exists to prove that natural eukaryotic endogenous circRNAs can be translated.

#### Other potential functions

CircRNAs have a protein bait function. Du et al. [40] found that circ-Foxo3 was distributed in the cytoplasm and was closely related to cell senescence. Circ-Foxo3 binds to the anti-aging proteins ID-1, E2F1, and HIF1α, and the anti-stress protein FAK to inhibit the effects of these proteins, thus promotes cell senescence. In addition, other studies have found that circ-Foxo3 binds to the cell cycle proteins, CDK2 and p21, to form the circ-Foxo3-p21-CDK2 ternary complex, thereby inhibiting the association between the CDK2 protein and cyclins A and E, and inhibiting the cell cycle [41].

Some of the circRNA-derived pseudogenes, such as circRFWD2, can be retrotranscribed and reinserted into the host genome, and this may change the structure of the genome, and thereby regulate gene expression [42].

### How circRNAs are detected

The technique of RNA sequencing is a transcriptomic research method based on second-generation gene sequencing. Early RNA sequencing uses the Poly(A) tail enrichment method to construct a library for sequencing. However, circRNAs lack the Poly(A) tail owing to the closed circular structure; thus, circRNAs have been largely rejected during library construction. Salzman et al. [20] first performed end-paired RNA sequencing using rRNA removal in multiple cell and tissue samples. They compared the generated read length and database data, combined those data with biochemical analysis, and found that circRNA was ubiquitous in cell transcription. Subsequently, a variety of algorithms were designed to define and quantify unknown circRNAs in RNA sequencing data; however, they presented differences in sensitivity and specificity [43].

Hansen et al. [44] evaluated five commonly used circRNAs prediction methods (circRNA_finder [45], find_circ [31], CIRCexplorer [25], CIRI [46] and MapSplice [47]). Among them, CIRI exhibited the highest sensitivity with the highest false-positive rate, whereas the opposite trend was observed for MapSplice. The circRNA_finder was the fastest detection. Furthermore, Zeng et al. [48] compared the performance of 11 circRNA detection tools and found that CIRI, CIRCexplorer and KNIFE [49] achieved better precision and sensitivity than the other methods, whereas NCLScan [50] and MapSplice had comparable precision with less favorable sensitivity. Conversely, Segemehl [51] together with find_circ and UROBORUS [52], exhibited the worst precision. Gao et al. [53] proposed a new algorithm, CIRI2, which encompasses the sensitivity of Segemehl and the false discovery rate of MapSplice, and outperformed the other eight methods commonly used for the prediction of circRNAs. In addition, they designed a new algorithm (CIRI-AS) to detect the internal composition of circRNAs in different species and found that circRNAs had alternative splicing and were expressed in a tissue- and developmental stage-specific manner.

Although RNA sequencing can detect a large number of circRNAs, they still need to be validated and confirmed by biochemical methods. Among those methods, RT-PCR is the simplest and fastest way to identify circRNAs. It amplifies and quantifies specific circRNAs with primers for the reverse splice site of the circRNAs. However, template conversion, trans-splicing, and unexpected gene duplication in the reverse transcription process result in false positives from RT-PCR [54]. The exoribonuclease, RNase R, progressively degrades RNA from its 3′ to 5′ end, and is used in combination with RT-PCR to identify the circRNAs. It is also used for purification of circRNAs in sequencing libraries and verification of sequencing results [43]. Northern blotting recognizes the circularized exonic sequence using designed probes, and RNase H is an endoribonuclease that is capable of cleaving RNA at RNA-DNA hybrids. Starke et al. [19] identified EcircRNAs’ closed loop structure by using Northern blotting with RNase H. In addition, RNA-sequencing combined with Northern blotting, can reveal the presence of alternative splicing of circRNAs in different cells, and further demonstrate that such circRNAs retaining part of the intron are the EIciRNAs [55]. In 2D-PAGE, linear RNA migrates along the diagonal of the 2D gel according to its size, whereas circRNA travels in an arc owing to its anomalous migration. Therefore, this method could be an enrichment, quantification and characterization method for circRNAs sequencing [56].

### CircRNAs and respiratory diseases

**Fig. 4:**
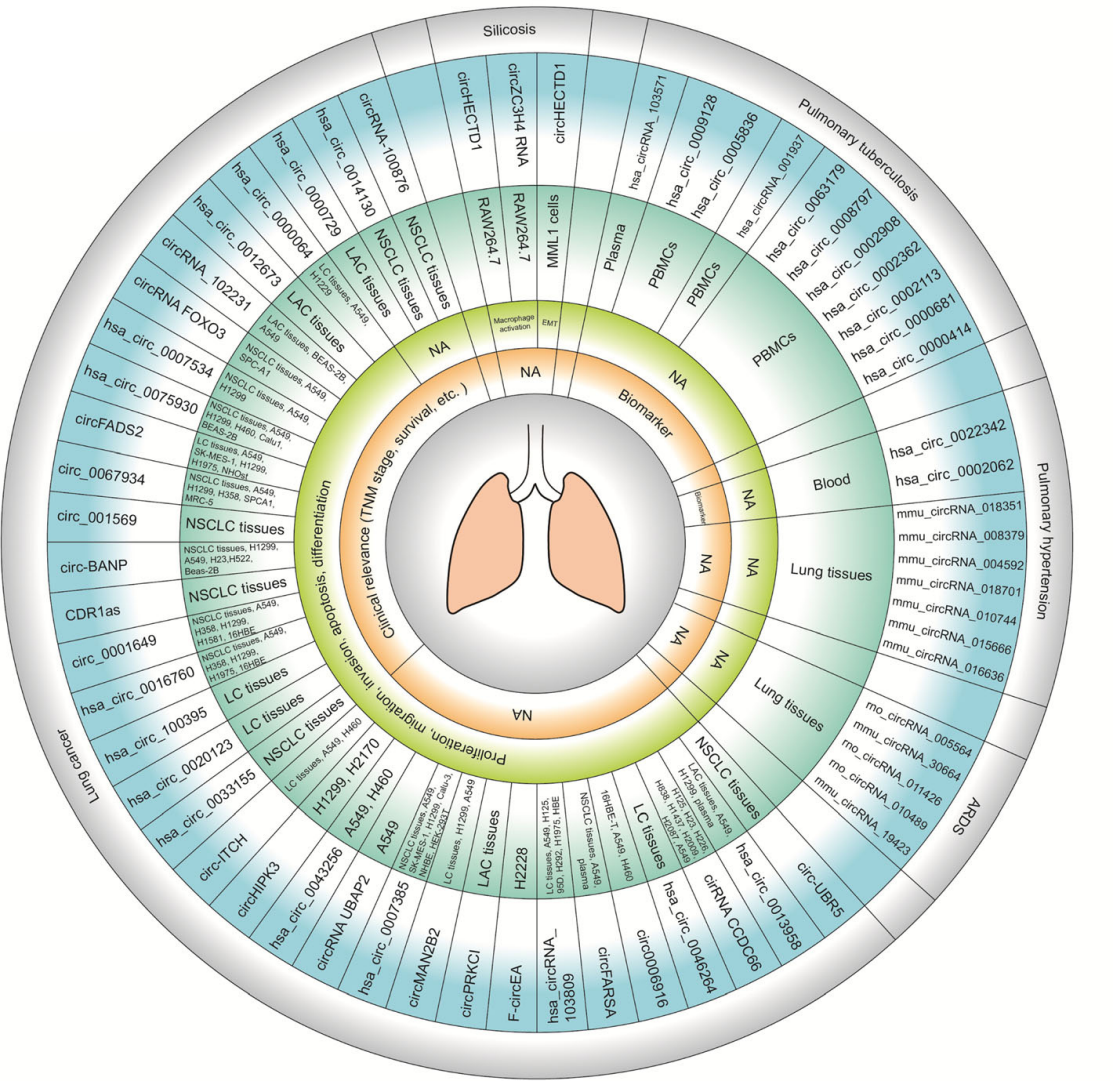
A summary of current studies on circRNAs in respiratory diseases. CircRNAs have been found to participate into several respiratory diseases, including lung cancer, ARDS, pulmonary hypertension, pulmonary tuberculosis and silicosis. NSCLC, non-small cell lung cancer; LAC, lung adenocarcinoma; LC, lung cancer; NA, no related studies; ARDS, acute respiratory distress syndrome

CircRNAs are reportedly involved in neoplastic and non-neoplastic respiratory diseases, suggesting their potential as a biomarker for respiratory diseases. So far, circRNAs have been identified in lung cancer, acute respiratory distress syndrome (ARDS), pulmonary hypertension, pulmonary tuberculosis (TB) and silicosis. Most of studies have focused on the role of circRNAs in lung cancer. We summarized all these studies to provide a more comprehensive understanding of the role of circRNAs in respiratory diseases (Fig. 4).

#### CircRNAs in lung cancer

Evidence suggests that circRNAs can enhance or suppress both the initiation and progression of lung cancer by repressing miRNAs, which are involved in various cell processes, including proliferation, differentiation, migration and carcinogenesis.

Some circRNAs, such as circRNA-ITCH and hsa_circ_0043256, reportedly play a protective role in lung cancer by up-regulating its parental gene ITCH expression and inactivating the Wnt/β-catenin pathway. Wan et al. [57] found that the expression of circ-ITCH was significantly reduced in lung cancer tissues, demonstrating that it functions as a sponge of multiple oncogenic miRNAs, including miR-7 and miR-214, and consequently inhibits lung cancer cell proliferation. The luciferase reporter assay, western blot, and RT-PCR further confirmed that circ-ITCH suppressed the activation of Wnt/β-catenin signaling by transcriptionally down-regulating β-catenin, c-Myc, and cyclin D1. Tian et al. [58] revealed that increased levels of hsa_circ_0043256, triggered by cinnamaldehyde, inhibited miR-1252 to up-regulate ITCH expression in H1299, YTMLC-90, and A549 cells, and inactivated the Wnt/β-catenin pathway, suggesting a possible anti-tumor role of this circRNA. In addition, Dai et al. [12] demonstrated that circ0006916 was significantly down-regulated in 16HBE-T, A549 and H460 cell lines and acted as a tumor suppressor gene by affecting cell cycle distribution and inhibiting cell proliferation. Further analysis showed that circ0006916 may act by sponging miR-522-3p and its downstream pleckstrin homology domain and leucine rich repeat protein phosphatase 1 (PHLPP1). Moreover, trinucleotide repeat-containing 6A (TNRC6A), an RBP, could enhance the production of circ0006916 by binding to recognition elements in the flanking intron. These findings add to our understanding of the upstream mechanisms of tumorigenesis for lung cancer.

Meanwhile, many oncogenic circRNAs are reportedly regulated in lung cancer. Levels of hsa_circ_0000064 is found increased in lung cancer tissues and cell lines (A549 and H1229) and its high expression is associated with the T stage, lymphatic metastasis, and TNM stage. Knockdown of hsa_circ_0000064 could suppress cell proliferation, induce apoptosis, alter the cell cycle, with increased expression of bcl-2, and decreased expression of cancer-related proteins involved in cell cycle (p21, CDK6, and cyclin D1) and cell apoptosis (caspase-3, caspase-9, and bax). Furthermore, expression levels of the migration and invasion-related proteins MMP-2 and MMP-9 are significantly reduced when hsa_circ_0000064 is knocked down, suggesting its possible role as an oncogenic circRNA and as a novel promising biomarker and therapeutic target for lung cancer [59]. Another circRNA, circRNA_100876, is significantly upregulated in comparison to paired adjacent non-tumorous tissues among the non-small cell lung cancer (NSCLC) tissues of 101 patients. This circRNA also shows a significant association with lymph node metastasis, advanced tumor staging, and shorter overall survival times of patients with NSCLC [60]. Very recently, Tan et al. [61] found that a fusion circRNA, named as F-circEA, which was generated from the EML4-ALK fusion gene with the function of promoting cell proliferation and migration, and could be detected in the serum of patients with NSCLC who are positive for EML4-ALK. This indicates that F-circEA may serve as a diagnostic marker of EML4-ALK-positive NSCLC.

#### CircRNAs in ARDS

As a major fatal respiratory disease, ARDS, a severe stage or type of acute lung injury (ALI), is characterized by refractory arterial hypoxemia and respiratory failure. Although many efforts have been done, there is still slow progression on the pathogenesis and therapy targets, which results in high morbidity and mortality of ARDS/ALI. Previous studies have shown that miRNAs such as miR-155 [62] and miR-17 [63] play a key role in the development and treatment of ALI. As miRNA sponges, circRNAs may help us from a new perspective to develop an effective therapy for ARDS. Wan et al. [64] firstly analyzed the circRNAs expression profiles of lung tissues from rat with LPS-induced ARDS, and identified 395 up- and 562 down-regulated circRNAs. Among them, 4 up-regulated circRNAs (mmu_circRNA_19423, rno_circRNA_010489, rno_circRNA_011426, mmu_circRNA_30664) and 1 down-regulated circRNA (rno_circRNA_005564) exhibited significant validity. Ye et al. [65] firstly identified 10 differentially expressed circRNAs in rat lungs following ALI caused by smoke inhalation. These changing circRNAs improve our understanding of molecular mechanisms and biological functions of circRNAs in ARDS/ALI, but more work, such as identifying their role and related mechanisms and further validating in patients with ARDS/ALI, are needed in the near future.

#### CircRNAs in pulmonary hypertension

Pulmonary hypertension refers to as a hemodynamic and pathophysiological state, in which pulmonary arterial pressure increases above a certain threshold. So far, numerous noncoding RNAs (lncRNAs and miRNAs) have been found to participate in the pathologic process of PH. CircRNAs, as a novel member of the non-coding RNA family, will be an important diagnostic and therapeutic target in PH. To the best of our knowledge, our team was the first to detect dysregulated circRNAs in the lungs of mice with hypoxia-induced PH by performing the circRNAs microarray. Among those detected, 23 significantly upregulated and 41 significantly downregulated circRNAs were identified. In addition, we used circRNA-miRNA-mRNA network analysis, and Gene Ontology (GO) and Kyoto Encyclopedia of Genes and Genomes (KEGG) analysis to formulate further insights into the related mechanisms and pathways. Our results suggested that dysregulated circRNAs may play key roles in the pathogenesis of hypoxia-induced PH, and can be potential targets for treatment [15]. Besides, Miao et al. [66] identified 351 (122 upregulated and 229 downregulated) differentially expressed circRNAs in chronic thromboembolic pulmonary hypertension (CTEPH). Furthermore, they found that upregulated circRNAs might function in CTEPH mainly by affecting the ribonucleotide biosynthetic process; whereas downregulated circRNAs may function mainly by regulating the cellular response to stress, DNA damage stimulus, and gene expression. Among them, hsa_circ_0002062 sponges has-miR-942-5p, which is mainly enriched in cancer-related pathways, and hsa_circ_0022342 sponges has-miR-940, which is mainly enriched in the ErbB signaling pathway. These two circRNAs may play key roles in CTEPH development, and their targeted regulation may be an effective approach treating CTEPH. These studies suggested a potential role of circRNAs in the development of PH, but are still in profiling dysregulated circRNAs and lack deeper function and mechanism research.

#### CircRNAs in pulmonary tuberculosis

Pulmonary tuberculosis, caused by Mycobacterium tuberculosis, remains a serious threat to public health owing to the delay in diagnosis and treatment. However, the current methods for the diagnosis of pulmonary TB are limited for their sensitivity and specificity. Thus, new biomarkers are urgently required to help early diagnosis and treatment for pulmonary TB. Zhang et al. [67] identified 170 dysregulated circRNAs in pulmonary TB, compared with healthy control and constructed ceRNAs. Their findings suggested an important role of circRNA-associated ceRNA-mediated gene regulation in the pathogenesis of pulmonary TB. Huang et al. [68] showed that hsa_circ_001937 present significant value in TB diagnosis (area under the curve = 0.873). It was correlated with TB severity, and served as a TB-specific signature circRNA, showing significantly increased levels in patients with TB, compared to those with pneumonia, chronic obstructive pulmonary diseases (COPD), and lung cancer. Zhuang et al. [69] found that hsa_circ_0005836 and has-circ-0009128 were significantly down-regulated in the peripheral blood mononuclear cells (PBMCs) of patients with active pulmonary TB, compared to healthy controls. GO-based enrichment and KEGG analysis showed that major biological functions of differentially expressed circRNAs were related to immune system activation, suggesting a correlation between TB infection and the activity of the immune system. Qian et al. [70] identified the differentially expressed circRNAs in PBMCs of TB patients and chose 7 circRNAs to construct circRNA-based TB index for each subject. The TB index was higher in TB patients than that in healthy control and the area under the receiver operating characteristic curve was 0.946 in validated groups. Further KEGG analysis showed that several pathways related to inflammation and bacteria invasion were upregulated in active TB patients. Furthermore, Yi et al. [71] determined the plasma circRNA expression profiles of active TB patients and found that hsa_circRNA_103571 exhibited a significant decrease in active TB patients. Further bioinformatics analysis showed that hsa_circRNA_103571 participated into regulation of actin cytoskeleton, T- and B-cell receptor signaling pathway. Previous studies have identified the diagnostic value of some circRNAs in TB infection, and further research may put emphasis on nontuberculous mycobacteria (NTB), which causes tuberculosis-like clinical symptoms and imaging performance, and thus easily results in misdiagnosis and mistreatment. Furthermore, most non-tuberculous mycobacteria are highly resistant to antituberculotic drugs and cause a large disease burden to society. In future, circRNAs may be a new biomarker tohelp physicians better distinguish non-tuberculosis infection from TB.

#### CircRNAs in silicosis

Silicosis is one of the most common, fastest-growing, and most serious types of pneumoconiosis, characterized by extensive nodular fibrosis in the lungs caused by long-term inhalation of large amounts of free silica dust. At present, circRNAs associated with silicosis have been subjected to in-depth study, mainly by Jie Chao’s research group. They [72] reported that circZC3H4 RNA sponged miR-212 and thereby relieved inhibition of the ZC3H4 protein by miR-212. Increased expression of circZC3H4 RNA and ZC3H4 upon SiO_2_ exposure can affect macrophages activation and their downstream effects on fibroblast proliferation and migration. Importantly, increased levels of the ZC3H4 protein have been confirmed in tissue samples from patients with silicosis, suggesting that ZC3H4 may be a potential therapeutic target for silicosis. Zhou et al. [14] found that the circHEZCD1/HECTD1 pathway participated in SiO2-induced macrophages activation and increased HECTD1 expression in patients with silicosis. Moreover, reduced levels of circHEZCD1 and increased levels of HECTD1 upon the exposure of SiO2 are regulated by ZC3H12A, a novel RBP. After that, Fang et al. [73] found that circHECTD1 might regulate the protein levels of its host gene, hectd1, through competition with its pre-mRNA. The circHECTD1/HECTD1 pathway, which promotes the endothelial–mesenchymal transition (EMT) in vitro following SiO2 exposure, may be a possible mechanism of fibrosis and new therapy target in patients with pulmonary silicosis.

## Conclusion and perspectives

As novel members of the RNA family with potential regulatory functions, circRNAs have been attracting increasing attention from several researchers on respiratory diseases. The potential application of circRNAs in respiratory diseases in future could be divided into two ways. One is to be new diagnostic and prognostic biomarkers for different respiratory diseases. The high stability and abundance of circRNAs in the peripheral blood and other body fluids make it to be a better biomarker compared to other RNA molecules like miRNAs and non-coding RNAs [74]. As mentioned in this review, several circRNAs have been identified to be clinical biomarkers for lung cancer, pulmonary hypertension and pulmonary tuberculosis. In future, more candidate circRNAs or circRNAs diagnostic panels will be detected to assist clinicians to diagnose different respiratory diseases.

Second is to be special therapeutic targets by modulating the expression of circRNAs in cells. The possible therapeutic mechanism of circRNAs is considered to act as miRNAs sponge or be translated to functional proteins [75]. At present, no clinical trials were conducted to explore the therapeutic effect of circRNAs in respiratory diseases. Hoverer, circRNAs have present its potential therapeutic value in different respiratory diseases, especially lung cancer, based on cell and animal models. The native protective circRNAs could be overexpressed via delivering circRNAs plasmids or viruses [76, 77]. Furthermore, with the advent of different methods for RNA circularization, personalized circRNAs could be designed and artificially synthesized according to the need of researchers or clinicians. Liu et al. [78] for the first time artificially synthesized a circRNA to inhibit the proliferation of gastric cancer cells via sponging miR-21. This study provided a new idea for the clinical application of circRNAs in respiratory diseases in future. Besides, the disease-promoting circRNAs could be knocked down by the exogenous delivery of special siRNA or CRISPR/Cas9-based gene editing to target the back-splice junction of circRNAs.

At present, study on the role of circRNAs in respiratory diseases is just beginning. The width and depth of recent studies are limited and there are still many challenges and chances for us to explore the role of circRNAs in respiratory diseases. Firstly, differentially expressed circRNAs have only been identified in lung cancer, ARDS, pulmonary hypertension, pulmonary tuberculosis and silicosis. The role of circRNAs in asthma, chronic obstructive pulmonary diseases, bronchiectasis and other pulmonary infectious diseases is completely unknown and required to be identified. Secondly, the molecular mechanisms of circRNAs in different respiratory diseases remain largely elusive. Most of studies only identified differentially expressed circRNAs using the circRNAs microarray or the next-generation gene sequencing, but the function of these circRNAs were not elucidated. Thus, more work is required to demonstrate the functions of these differentially expressed circRNAs in respiratory diseases-related cell and animal models in future. Finally, the circRNAs diagnostic panels and therapeutic methods targeting circRNAs need to be developed. The sensitivity and reliability of circRNAs as biomarkers are required more clinical validation in different respiratory diseases. Prior to clinical application of circRNAs-based therapeutic methods, several problems including side effects, therapeutic efficiency, delivery strategies and ethical issues, should be resolved.

Taken together, circRNAs have become a major focal point of research studies on human diseases, and they represent a promising prospect with high organ specificity in the respiratory disease diagnosis, treatment, and prognosis in the near future.

## Abbreviations

ALI: Acute lung injury
ARDS: Acute respiratory distress syndrome
ceRNAs: competitive endogenous RNAs
CircRNAs: Circular RNAs
CiRNAs: Intronic circRNAs
COPD: Chronic obstructive pulmonary diseases
CTEPH: Chronic thromboembolic pulmonary hypertension
EcircRNAs: Exonic circRNAs
EIciRNAs: Exon-intron circRNAs
EMT: Endothelial–mesenchymal transition
GO: Gene ontology
IRES: Internal ribosome entry sites
KEGG: Kyoto encyclopedia of genes and genomes.
miRNAs: microRNAs
MREs: MiRNA response elements
NSCLC: Non-small cell lung cancer
NTB: Nontuberculous mycobacteria
ORF: Open reading frame
PBMCs: Peripheral blood mononuclear cells
PH: Pulmonary hypertension
PHLPP1: Pleckstrin homology domain and leucine rich repeat protein phosphatase 1
pre-mRNAs: precursor mRNAs
RBPs: RNA binding proteins
snRNPs: small nuclear ribonucleoproteins
TB: Tuberculosis
